# Dose-Dependent Variation of Synchronous Metabolites and Modules in a Yin/Yang Transformation Model of Appointed Ischemia Metabolic Networks

**DOI:** 10.3389/fnins.2021.645185

**Published:** 2021-08-20

**Authors:** Yifei Qi, Niwen Zhou, Qing Jiang, Zhi Wang, Yingying Zhang, Bing Li, Wenjuan Xu, Jun Liu, Zhong Wang, Lixing Zhu

**Affiliations:** ^1^Institute of Basic Research in Clinical Medicine, China Academy of Chinese Medical Sciences, Beijing, China; ^2^Xiyuan Hospital, Institute of Geriatrics, China Academy of Chinese Medical Sciences, Beijing, China; ^3^Center for Statistics and Data Science, Beijing Normal University at Zhuhai, Zhuhai, China; ^4^Global Business Services, International Business Machines Corporation, Shanghai, China; ^5^Dongzhimen Hospital, Beijing University of Chinese Medicine, Beijing, China; ^6^Institute of Chinese Materia Medica, China Academy of Chinese Medical Sciences, Beijing, China; ^7^School of Life Sciences, Beijing University of Chinese Medicine, Beijing, China; ^8^Department of Mathematics, Hong Kong Baptist University, Hong Kong, China

**Keywords:** multitarget interaction, synchronous module, module pharmacology, dose-dependent relationship, cerebral infarction

## Abstract

**Aim:**

Chinese medicine Danhong injection (DHI) is an effective pharmaceutical preparation for treating cerebral infarction. Our previous study shows that DHI can remarkably reduce the ischemic stroke-induced infarct volume in a dose-dependent manner, but the pharmacological mechanism of the DHI dose-dependent relationship is not clear. Therefore, the dose-dependent efficacy of DHI on cerebral ischemia and the underlying mechanisms were further investigated in this study.

**Methods:**

A middle cerebral artery occlusion (MCAO) model was established and the rats were randomly divided into six groups: sham, vehicle, DHI dose-1, DHI dose-2, DHI dose-3, and DHI dose-4. Forty-one metabolites in serum were selected as candidate biomarkers of efficacy phenotypes by the Agilent 1290 rapid-resolution liquid chromatography system coupled with the Agilent 6550 Q-TOF MS system. Then, the metabolic networks in each group were constructed using the Weighted Correlation Network analysis (WGCNA). Moreover, the Yang and Yin transformation of six patterns (which are defined by up- and downregulation of metabolites) and synchronous modules divided from a synchronous network were used to dynamically analyze the mechanism of the drug’s effectiveness.

**Results:**

The neuroprotective effect of DHI has shown a dose-dependent manner, and the high-dose group (DH3 and DH4) effect is better. The entropy of the metabolic network and the Yin/Yang index both showed a consistent dose–response relationship. Seven dose-sensitive metabolites maintained constant inverse upregulation or downregulation in the four dose groups. Three synchronous modules for the DH1–DH4 full-course network were identified. Glycine, N-acetyl-L-glutamate, and tetrahydrofolate as a new emerging module appeared in DH2/DH3 and enriched in glutamine and glutamate metabolism-related pathways.

**Conclusion:**

This study takes the DHI metabolic network as an example to provide a new method for the discovery of multiple targets related to pharmacological effects. Our results show that the three conservative allosteric module nodes, taurine, L-tyrosine, and L-leucine, may be one of the basic mechanisms of DHI in the treatment of cerebral infarction, and the other three new emerging module nodes glyoxylate, L-glutamate, and L-valine may participate in the glutamine and glutamate metabolism pathway to improve the efficacy of DHI.

## Introduction

Understanding how complex interactions among components give rise to emergent properties of biological systems has become a controversial issue for researchers (Kitano, 2002). When truly describing a biological network, holistic and dynamics are two important features (Tyson et al., 2011). Dynamics study is generally conducted with a series of ordinary differential equations. Although some methods do not require knowledge of the kinetic parameters, such as Boolean network modeling, important components for identifying the construction of an interaction network must be based on a large amount of prior hybrid knowledge data (Bloomingdale et al., 2018). Module pharmacology is a useful tool for decomposing a complex network. In our previous study, dynamic behaviors were evaluated according to several entropies to explore the borderline transformation. This simply entailed comparing the static modules to infer the flexible state of the dynamic modules (Yu et al., 2016). Synchronization is a special form of dynamic change, and it is ubiquitous in biological systems, such as transportation systems, chemical reactions, metabolic processes, and neuroscience domains (Vicsek and Zafeiris, 2012). This allows us to see the overall structure of modular mechanisms by using a top-down, data-driven approach that identifies differentially expressed components from the metabolomics data.

Stroke is the second leading cause of death and the main cause of long-term disability in those who survive. Approximately 16 million first-ever strokes occur each year in the world, resulting in nearly 6.2 million deaths (Mendis et al., 2011). With our growing understanding of complex pathological mechanisms, both multiple protein targets mediated the specific efficacy (Yıldırım et al., 2007; Chan and Loscalzo, 2012) and the off-target side effects [stemming from specific druggable protein targets, such as the thrombolytic agents alteplase and tenecteplase, which cause proteolysis of apolipoprotein A-I and potentially result in the transient impairment of HDL atheroprotective functions (Gomaraschi et al., 2013)] provide a warning that we should be seeking feasible prevention and treatment strategies.

Danhong injection (DHI) is a medicinal preparation based on two reputed Chinese herbal remedies, Salviae Miltiorrhizae and Flos Carthami. It is characterized by multiple compounds and multiple biological targets, and it has attracted extensive attention for its ability to treat cardiovascular and cerebrovascular diseases due to its moderate treatment effect and low side effects when using a combination of different drugs and dose compatibility. Recent studies show that DHI can remarkably reduce the ischemic stroke-induced infarct volume in a dose-dependent manner (Jiang et al., 2015; Zhang et al., 2017), but the pharmacological mechanism of the DHI dose-dependent relationship is not clear, although anti-coagulatory, anti-inflammatory, anti-oxidant, anti-apoptotic, vasodilatory, and angiogenesis-promoting actions have been demonstrated (Feng et al., 2019). Therefore, in this work based on the study of dynamics, we attempted to find synchronous modules composed of multi-coupling targets to further reveal the dose-dependent pharmacological mechanisms of DHI treatment for cerebral ischemia, so that expectation provides deeper analysis for stroke treatment.

## Materials and Methods

### MCAO Model and Drug Treatments

All procedures of experiments were reviewed and approved by the Ethics Review Committee for Animal Experimentation, China Academy of Chinese Medical Sciences, and it was implemented according to the national laws of China as well as local guidelines. A total of 60 Sprague–Dawley male rats (weighing 180–220 g) were used in the study. All rats were randomized into six groups: sham, vehicle, DHI dose-1 (DH1, 1 ml/kg, iv), DHI dose-2 (DH2, 2.5 ml/kg, iv), DHI dose-3 (DH3, 5 ml/kg, iv), and DHI dose-4 (DH4, 10 ml/kg, iv), with 10 animals for each group.

The rat transient middle cerebral artery occlusion (MCAO) model was induced as described elsewhere (Guo et al., 2015; Zhu et al., 2015; Wenjuan et al., 2018). Briefly, rats were anesthetized with 10% chloral hydrate (4 ml/kg, i.p.), and a monofilament with a silicon-coated tip was inserted into the internal carotid artery and advanced until it obstructed the middle cerebral artery. The blood circulation was blocked for 1 h and then restored by removing the monofilament. Sham-operated rats received the same manner as the MCAO rats without ischemia, the external carotid artery was surgically prepared for the insertion of the filament, but the monofilament was not inserted. All animal experiments were conducted in accordance with the ethical principles of animal use and care.

Then, the drug was injected through the caudal vein with 1 ml/kg euvolemia, which was diluted by physiological saline of varying amounts according to the concentrations in the different groups once daily for three continuous days. The sham and vehicle groups were injected with the same doses of saline. DH injections were supplied by ShanXi BuChang Pharmaceutical Corp., Ltd.

Blood samples were taken from the abdominal aorta on the third day after anesthetization, and the brain was removed rapidly.

### Evaluation of Pharmacological Effects

To evaluate the protective effect of DHI against cerebral ischemia, the cerebral infarct volume, brain weight index, and neurological deficit score were measured.

Triphenyltetrazolium chloride (TTC) staining was used to determine infarction volume. Two-millimeter-thick slices of the cerebrum were immersed in a 2% TTC solution at 37°C for 30 min. Infarct area was measured using the Scion Image program, and the areas of ischemic lesions were calculated using [contralateral hemisphere volume −(ipsilateral hemisphere volume − measured injury volume)].

After 36 h of reperfusion, the brains were swiftly removed. The brain samples and bodies of the rats were weighed immediately. The brain weight index was expressed as brains mass (g)/total rat mass (g).

Neurological deficit scoring (NDS) was measured at first, second, and third after reperfusion by an investigator who was not involved in the study. Consciousness, breathing, smell, vision and hearing, reflexes, motor function, overall activity, orientation, and presence of seizures were scored, according to the neurological deficit score system [0–4 scale; 0, no motor deficit (normal); 1, forelimb weakness and torso turning to the ipsilateral side when held by tail (mild); 2, circling to the contralateral side but normal posture at rest (moderate); 3, unable to bear weight on the affected side at rest (severe); and 4, no spontaneous locomotor activity or barrel rolling (critical)] adapted from a previous study.

### Targeted Metabolomics Analysis

Serum samples were collected for targeted metabolomics analysis based on an Agilent 1290 rapid-resolution liquid chromatography system coupled with an Agilent 6550 QTOF/MS system, and the metabolite extraction process was performed as described in previous research with little modification (Dettmer et al., 2011). The targeted metabolomics analysis was used to determine the key nodes in the cerebral ischemia modules adapted in this work, which were described in a previous study (Wenjuan et al., 2018).

### The Network Entropy in Different Groups

Metabolite expression was quantitively identified by the HPLC system for each group. Based on the metabolite expression data, the metabolic networks were constructed using the WGCNA R package (Langfelder and Horvath, 2008). A matrix of pairwise correlations was constructed between all pairs of metabolites across the measured samples using appropriate soft thresholding for each group. The thresholds were selected when the network achieved the best scale-free topology criterion (Langfelder et al., 2008).

After construction of the metabolic networks for the six groups, we characterized them in terms of network entropy, which can be decomposed into contributions from individual nodes (Tan and Wu, 2004; Li et al., 2009):

$${\text{I}}_{\text{i}\;=\frac{{\text{k}}_{\text{i}}}{\sum_{\text{i}=1}^{\text{N}}{\text{k}}_{\text{i}}}}$$

where I_*i*_ is the importance of node i, N is the number of nodes in the network, and k_*i*_ is the degree of node i. The network structure entropy is defined as follows:

(1) $$\text{E}=-\sum_{i=1}^{N}I_{i}\,l\,n\,I_{i}$$

### The Yin and Yang Metabolite Expressional Transformation Patterns

A method that integrates all metabolite data was proposed to explore the equilibrium state between Yin (downregulated expression) and Yang (upregulated expression). All of the metabolites among the different groups were compared. First, we compared the vehicle group with the sham group. Then, we compared the DHI groups with the vehicle group. The six metabolites’ expression transformation patterns were defined: The metabolites with expression levels that changed from negative to positive were defined as positive transformation (PT). Those with expression levels that changed from positive to negative, on the contrary, were defined as negative transformation (NT). The metabolites with expression that changed from a low positive value to a higher positive value were defined as positive add (PA). Those metabolites with expression values that remained negative yet demonstrated an increasing trend were defined as negative add (NA). Those with high negative values that decreased to lower negative values were named as negative reduce (NR). Metabolites with expression levels that remained positive despite a decreasing trend were defined as positive reduce (PR). Among these, PT, PA, and NA were regarded as yang because of their upregulation character. By comparison, NT, PR, and NR were regarded as yin because of their downregulation character.

All of the metabolites were classified into the above six patterns. The cumulative summation of each class of metabolites was denoted as PT^*a*^, PA^*a*^, NA^*a*^, NT^*a*^, PR^*a*^, and NR^*a*^, respectively. Subsequently, the Yin/Yang ratio and difference were observed. Because PT and NT reflect qualitative changes, a specific comparison between PT^*a*^ and NT^*a*^ was performed. The formulas are as follows:

SeparateRatio=PT/aNTa

$$\text{Separate Difference}={\text{PT}}^{\text{a}}-{\text{NT}}^{\text{a}}$$

Overallratio=(PT+aPA+aNA)a/(NT+aPR+aNR)a

OverallDifference=(PT+aPA+aNA)a-(NT+aPR+aNR)a

### Constructing Target Networks and Identifying Synchronous Modules in Different Groups

In this section, we used Kendall’s coefficient of concordance, or Kendall’s Tau, to evaluate the association between any two different metabolites. Recall that the definition of Kendall’s Tau is as follows:

$$P_{r}\,\left(P_{1}\,{\mathrm{andP}}_{2}\,\mathrm{are}\,\mathrm{concordant}\right)-P_{r}\,\left(P_{1}\,{\mathrm{andP}}_{2}\,\mathrm{are}\,\mathrm{discordant}\right)$$

with⁢τ∈[-1, 1]

In the above formula, *P*_1_ and *P*_2_ are two independent points distributed as (*X*,*Y*) and the probability *P*_*r*_(⋅) is defined on ℱ with ℱ being the distribution function of bivariate random vectors (*X*,*Y*). The two points *P*_1_ and *P*_2_ on the plane are said to be concordant if joining them produces a positive slope; they are discordant if the slope is negative. Thus, τ=0, implying that *X* and *Y* are independent, and τ±1 means that *Y* = *f*(*X*) for some monotone function *f*. The sample estimator of τ is

tn=(n2)-1⁢∑(n,2)t⁢(Pi,Pj).

Here,

$$t\,\left(p_{1},p_{2}\right)=s\,g\,n\,\left(X_{1}-X_{2}\right)\,\left(Y_{1}-Y_{2}\right)=\{\begin{array}{c} 1\,\mathrm{if}\,P_{1}\,\mathrm{and}\,P_{2}\,\mathrm{are}\,\mathrm{concordant};\\ -1\,\mathrm{if}\,P_{1}\,\mathrm{and}\,P_{2}\,\mathrm{are}\,\mathrm{discordant}, \end{array}$$

and sgn(u) is the sign function.

Thus, the Kendall’s Tau estimator *t_n_* reflects the proportion of concordant pairs of points in a sample *P*_1_,,*P*_*n*_ minus the proportion of pairs of points that are discordant. We estimated Kendall’s Tau between each two distinct set of metabolites.

To quantitatively describe the synchronization of pairwise nodes, we determined an absolute value greater than 0.5 as a threshold value. We selected pairwise nodes that were in accordance with the threshold for network construction, and an open-source software, Cytoscape 3.6.1, was used to visualize the networks. To identify the synchronous pairwise nodes with a higher degree of synchronization, we defined synchronous pairwise nodes and asynchronous pairwise nodes as those with correlation coefficients greater than 0.7 or less than −0.7, respectively. Functional modules were identified using the Molecular Complex Detection (MCODE) method (Bader and Hogue, 2003).

Kendall’s Tau was estimated between each two distinct metabolites, which were reconstructed from the different groups. If the module had been full-course synchronous during DH1, DH2, DH3, and DH4, it was called DH1–DH4; otherwise, it was called a partial synchronous module, such as DH1/DH2, DH3/DH4, or DH2/DH3. In this process, there existed both corotating changes and reverse changes, which were defined as synchronization and anti-synchronization, respectively. The synchronous module and anti-synchronous module represent the synergistic and antagonistic relationships between the metabolites, respectively.

### Enrichment Analysis of Gene Ontology (GO) Categories and KEGG Pathways

The pathway enrichment analysis of synchronous modules was employed on the MetaboAnalyst^1^ to evaluate the most relevant biological roles involved in the conditions. This analysis was conducted using a modified Fisher’s exact test. We selected all pathways that were significant with a *p*-value < 0.05 after correcting for multiple-term testing.

## Results

### Dose-Dependent Relationship of DHI on Acute Cerebral Ischemia

**FIGURE 1:**
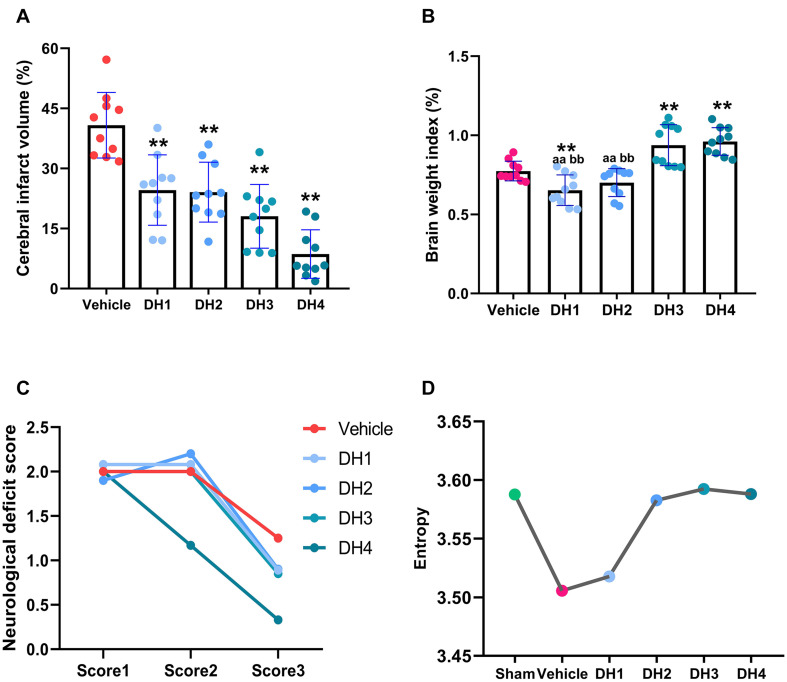
Dose-dependent relationship of DHI in the treatment of cerebral ischemia. **(A)** The effect of DHI on cerebral infarct volume (one-way ANOVA). **(B)** Changes in the body weight index in each group (non-parametric test). **(C)** Changes in neurological deficit score in each group. **(D)** The metabolic network entropy of the different groups. Values are depicted as mean ± SD, *n* = 10 for the sham, vehicle, and each of the DHI groups. **p < 0.01 compared with vehicle. ^*aa*^*p* < 0.01, compared with DH4. ^*bb*^*p* < 0.01, compared with DH3.

Compared with the vehicle group, DHI significantly reduced the infarction volume, increased brain weight index, and improved the neurological deficit score with a dose-dependent relationship. Overall, efficacy increased with increasing dose (Figures 1A–C; Guo et al., 2015; Zhu et al., 2015; Wenjuan et al., 2018). In terms of the brain weight index, the slope of the linear fit between the DHI multi-groups was 0.89. Compared with vehicle, DH3 and DH4 showed a significant difference, but no significant difference was found between vehicle, DH1, and DH2. Between the multiple DHI groups, DH3 and DH4 were significantly different from DH1 to DH2, but no significant difference was found between DH3 and DH4 or between DH1 and DH2. Therefore, we divided the four DHI dose groups into a low-dose category (DH1 and DH2) and a high-dose category (DH3 and DH4). The high-dose category was significantly more effective than the low-dose group.

### The Serum Metabolic Profiles and Network Entropy in Different Dose Groups

Based on the fatty acid/amino acid profiling and fusing maps of comprehensive serum metabolome through our previous studies, 41 metabolites were selected as candidate biomarkers related to efficacy of DHI (Wenjuan et al., 2018). Detailed information on these compounds is shown in Supplementary Table 1.

As illustrated in Figure 1D, compared with the sham group, the network entropy value decreased from 3.59 to 3.51 for the vehicle group. This result was reversed when DHI were given. With the DHI concentration increasing, the entropy values also gradually increased, up until it reached the normal level, where it remained relatively constant. Specifically, DH1 entropy value was 3.52, which was slightly higher than the vehicle group and increased by 12.5%. With an increasing dose, the entropy value of the metabolic network gradually increased. In the DH2 group, this value gradually returned to the normal level, which was increased by 94%. The DH3 and DH4 groups tended to maintain a relatively stable, high level. In general, this result is consistent with the trend of the efficacy measured by animal experiments. It also indicated that the 41 metabolites that we selected can represent efficacy phenotypes at a molecular level.

### Qualitative Analysis of the Yin/Yang Characteristics of DHI Efficacy Metabolites

**FIGURE 2:**
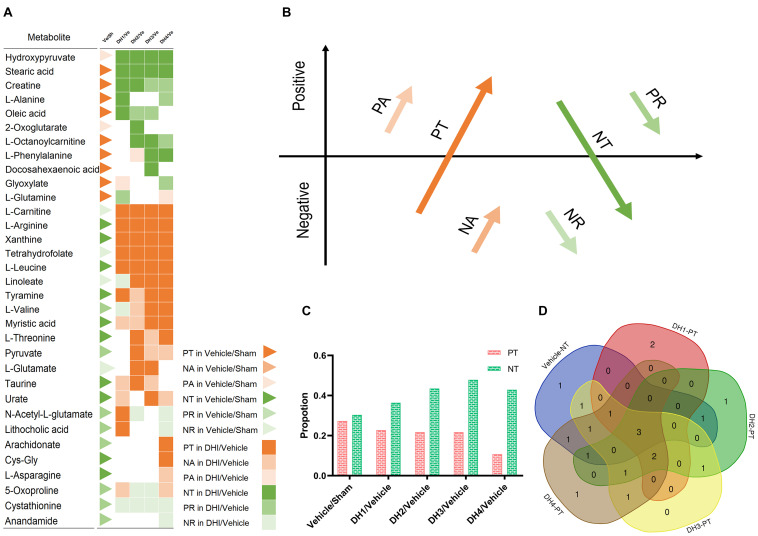
The Yin and Yang metabolite expressional transformation patterns. **(A)** Scheme of elucidating the six metabolites’ expression transformation patterns: positive transformation (PT), negative add (NA), positive add (PA), negative transformation (NT), positive reduce (PR), and negative reduce (NR). Red color indicates the Yang (PT, PA, and NA) and green color indicates the Yin (NT, PR, and NR). **(B)** Visualization of the six patterns of transformation between the 41 metabolite biomarkers in the group comparisons. The triangle indicates the comparison between the vehicle and sham group, and the squares indicate the comparison result between the DHI groups and the vehicle group. The color labels, which indicate the six transforming force patterns, are the same as those in **(A)**. **(C)** The proportion of PT or NT in the total six transformation patterns. **(D)** The Venn diagram of NT in the vehicle and other five patterns in the DHI groups.

Based on the expression level, 41 metabolite biomarkers in each group were classified into the six patterns of transformation (Figure 2A), as described in section “The Yin and Yang Metabolite Expressional Transformation Patterns” (Figure 2B). Generally, Yin dominates in the cerebral infarction state, which accounts for approximately 66.7% of the total metabolites. This was entirely reversed in the DHI groups which manifested by changes in these metabolites’ expression from Yin to Yang. The same result was found in the Yang aspect. In particular, seven metabolites constantly maintained the PT or NT reverse trend, including hydroxypyruvate, stearic acid, L-carnitine, L-arginine, xanthine, tetrahydrofolate, and L-leucine. Some metabolites, such as myristic acid, gradually reversed from NA to PT. With the dose increasing, some dose-insensitive metabolites only changed into PT in DH4, such as arachidonate and Cys-Gly. Two metabolites, i.e., cystathionine and anandamide, maintained Yin status and did not reverse. In brief, PT and NT transformation power are dominant whether in the pathologic or DHI treatment process. According to the dose–effect interaction, the metabolites’ transformation manifested as PT gradually increased and NT gradually decreased (Figure 2C), i.e., the Yin-to-Yang conversion.

### Quantitative Analysis of Six Transforming Force Patterns Related to the DHI Dose-Dependent Mechanism

**FIGURE 3:**
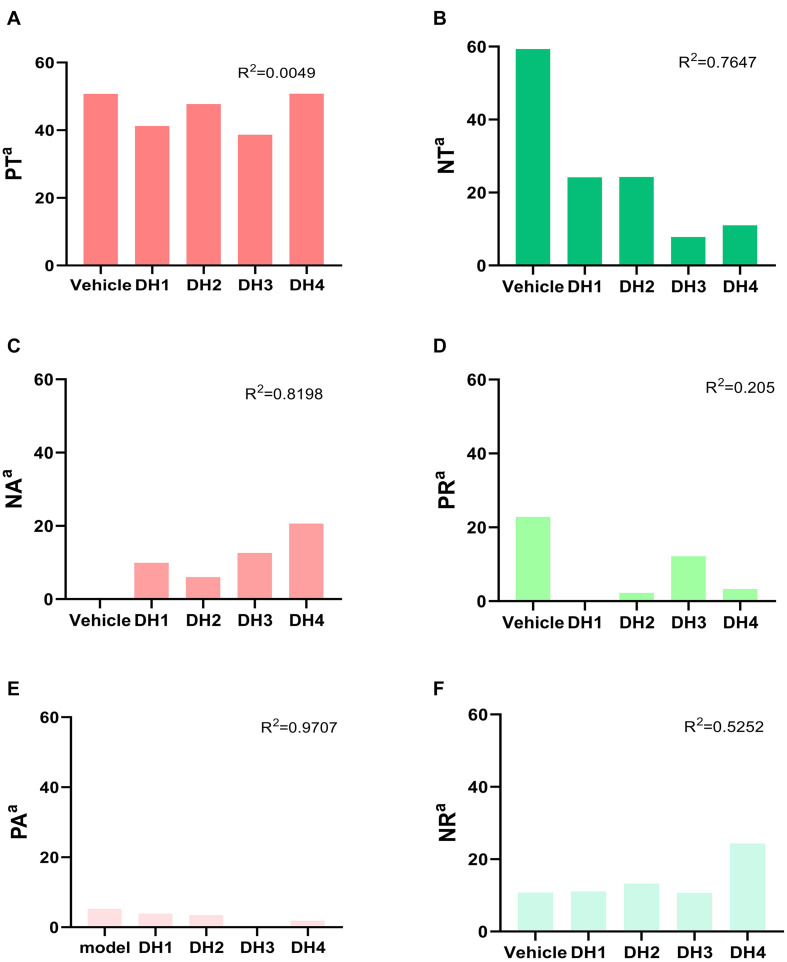
Cumulative summation of six transforming force patterns related to the DHI dose-dependent mechanism. **(A–F)** are the cumulative sum of PT, NT, NA, PR, PA, and NR in different groups, respectively.

To describe the specific trends of six transforming force patterns, we calculated the cumulative sum of PT, NT, NA, PA, PR, and NR in the different DHI dose groups, respectively. The details are shown in Figure 3. From a quantitative perspective, transformation power represented by PT and NT was still dominant. Compared with the vehicle group, the number of NT metabolites constantly decreased as the DHI dose increased, and the correlation coefficient was 0.76 (Figure 3B). Moreover, the number of PT metabolites was maintained at a high proportion in multiple dose groups (Figure 3A). Although their inclusive metabolites were different, there was no overlap of PT metabolites among the multiple DHI dose groups and the vehicle group (Figure 2D). From the vehicle group to the DHI treatment groups, the number of NA and PR metabolites showed a dose-dependent growth associated with increases in DHI concentration (Figures 3C,D), and the correlation coefficients were 0.82 and 0.21, respectively. There was no correlation among the vehicle group and multiple DHI dose groups, because of the relatively few numbers of PA metabolites (Figure 3E). NR in the DH1, DH2, and DH3 groups were similar to the vehicle group, but in the DH4 group, it was twice as many as the vehicle group (Figure 3F). Generally, this result shows that inhibiting the downregulation power of Yin was mainly contributing to the efficacy of DHI treatment.

### The Overall Yin/Yang Ratio or Difference Related to DHI Treatment

**FIGURE 4:**
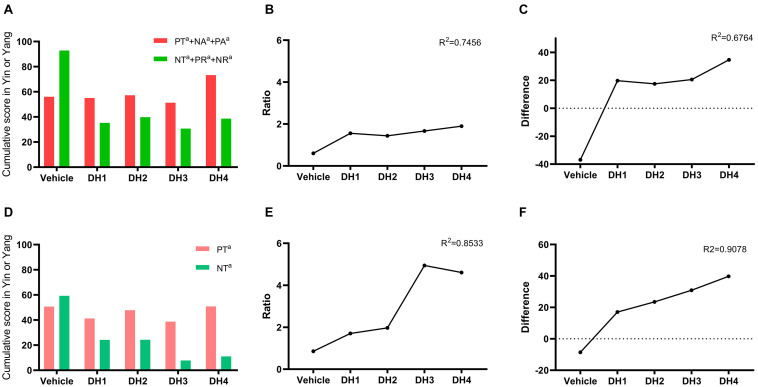
The ratio and difference between Yin/Yang related to DHI treatment. **(A)** Cumulative summation of total Yin (PT+NA+PA) and Yang (NT+PR+NR) related to DHI treatment. **(B,C)** The total Yin (PT+NA+PA) and Yang (NT+PR+NR) ratio and difference related to DHI treatment, respectively. **(D)** Cumulative summation of partial Yin (PT) and Yang (NT) related to DHI treatment. **(E,F)** The partial Yin (PT) and Yang (NT) ratio and difference related to DHI treatment, respectively.

As described in section “The Yin and Yang Metabolite Expressional Transformation Patterns,” we calculated the ratio and difference of the Yang/Yin cumulative sum. First, PT, NT, NA, PA, PR, and NR were divided into two Yin/Yang categories, and their differences were compared. The results show that the difference between Yin and Yang gradually increased, and the equilibrium state was broken when DHI was introduced (Figures 4A–C). Second, since PT and NT play a dominant role, we compared these two powers in isolation, and the same trends were obtained. Compared with the PT/NT conversation, the overall Yin/Yang comparison had higher linear fit scores on the ratio and difference, i.e., 0.85 and 0.91, respectively (Figures 4D–F).

### Dynamic Synchronous Module of the DHI Dose–Response Mechanism

In terms of synchronization node pairs, we found that a metabolite pair—oleic acid and hexadecanoic acid—always exists in dose change, and only the negative control node pair of octadecatrienoic acid and L-threonine appears in the conversion dose group.

**FIGURE 5:**
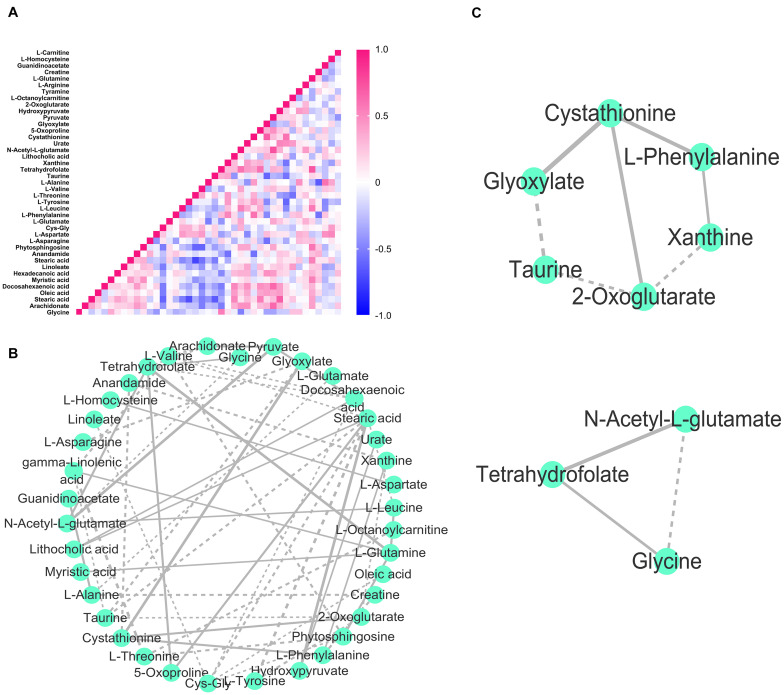
Module analysis in the DH2/DH3 synchronous network. **(A)** Heat map of Kendall’s coefficient between metabolites in DH2/DH3. The colored boxes on the right indicate the relative coefficient of the corresponding metabolite. **(B)** DH2/DH3 synchronous network constructed by Kendall’s coefficient of concordance. Solid lines indicate positive synchronization, and dashed lines indicate negative synchronization between nodes. The thickness of the line is proportional to Kendall’s correlation coefficient. **(C)** Two synchronous modules divided in the DH2/DH3 network.

In the synchronous network, we identified three modules of the DH1–DH4 full-course synchronous network and two, three, and two modules of DH1/DH2, DH3/DH4, and DH2/DH3 synchronous networks, respectively, using the MCODE algorithm (Figure 5 and Supplementary Figures 1, 3, 6). Based on pathway enrichment analysis, the three full-course synchronized modules were enriched to specific signal pathways of linoleic acid metabolism, fatty acid biosynthesis, and phenylalanine, tyrosine, and tryptophan biosynthesis, and so on (Supplementary Figure 5).

**FIGURE 6:**
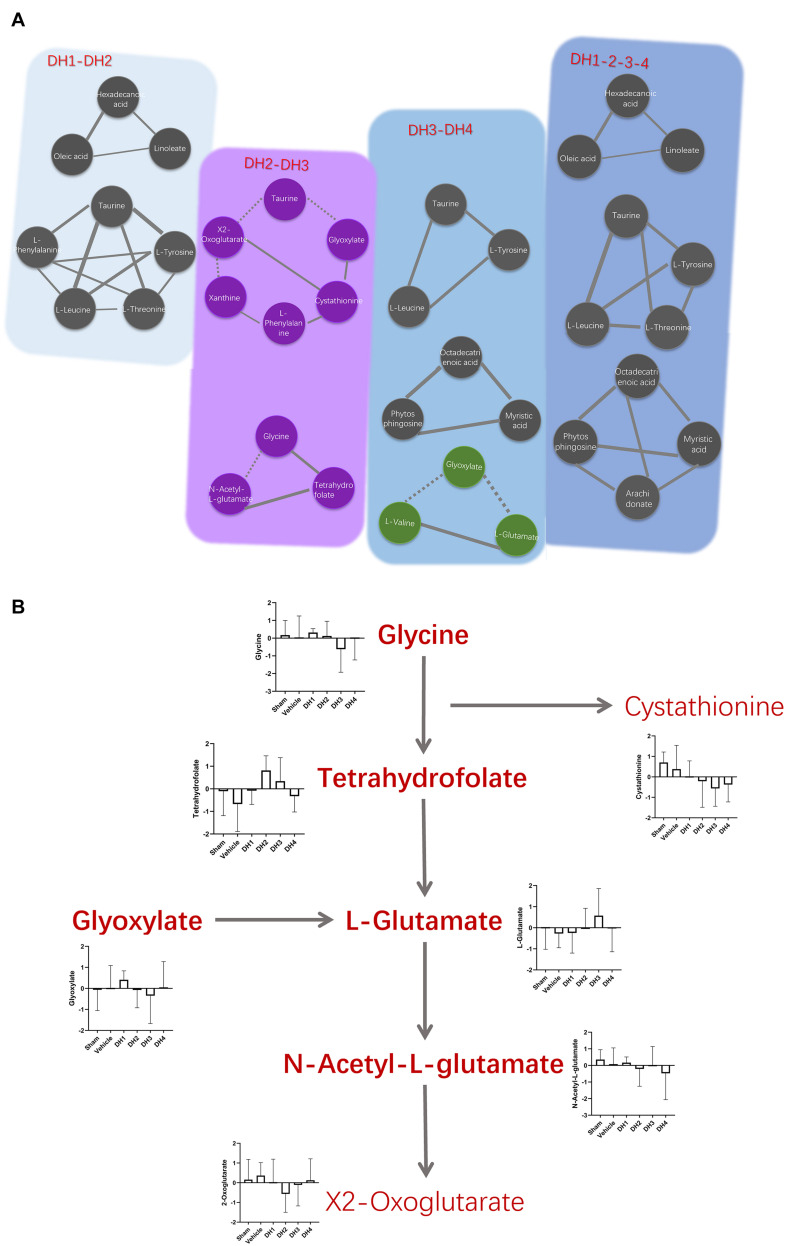
Variation of the synchronous modules on cerebral ischemia and the regularity of DHI. **(A)** Comparison of synchronous modules in different situations. Solid and dashed lines represent positive and negative correlation coefficients, respectively, the same as described above. Purple and green nodes represent specific modules in DH2/DH3 and DH3/DH4 synchronous networks, respectively. **(B)** The dynamic balance system of neurological amino acids in different states. The histogram shows the expression level of each metabolite in multiple groups.

With different DHI treatment doses, similar and individual synchronous modules may reveal molecular mechanisms. By comparing the synchronous modules in the DH1/DH2 and DH3/DH4 networks, a conservative allosteric module was obtained, which contained three constant nodes: taurine, L-tyrosine, and L-leucine (Figure 6A). It may represent the basic mechanisms of DHI in the treatment of cerebral infarction. Meanwhile, there are three unique synchronous modules obtained. One was composed of hexadecanoic acid, oleic acid, and linoleate in the DH1/DH2 synchronous network (Supplementary Figure 3). The other two modules in the DH3/DH4 synchronous network were composed of octadecatrienoic acid, phytosphingosine, myristic acid, and glyoxylate, L-valine, and L-glutamate (Supplementary Figure 6). More importantly, a new emerging module of the DH3/DH4 synchronous network was found, which was composed of glyoxylate, L-glutamate, and L-valine. The KEGG enrichment analysis of this specific module indicated that it was mainly involved in the glutamine and glutamate metabolism pathway (Supplementary Figures 4, 7). As for the DH2/DH3 synchronous network, which represents the low to high DHI dose transformation, two synchronous modules were obtained, and they were unique to DH2/DH3 (Figure 6A). These modules mainly participated in the regulation of glycine, serine, threonine, glyoxylate, and dicarboxylate metabolism (Supplementary Figure 5).

## Discussion

“Yin Ping Yang Bi” is symbolic of body health in TCM (Jiehua, 2012). It is an orderly and stable state of the body, in which material, energy, and information circulation can achieve equilibrium but not balance (López et al., 2016). In our study, Yin and Yang represent downward and upward trends of metabolite expression, respectively, and entropy value represents the flow of information at the metabolic level. As is known, there are two opposing states in the field of thermodynamics. One is the equilibrium state, which is a disordered state with a large degree of entropy; the other is the ordered steady state, with low entropy. Cerebral infarction is caused by interruption of blood flow to brain tissue, ischemia, hypoxia, and subsequent generation of a series of metabolic changes in cerebrum. Therefore, we assumed that cerebral infarction is a non-equilibrium state with a series of pathological changes, such as regional cerebral blood flow interruption or increase, oxidative factor chemotaxis, and oxidative stress response. DHI treatment of cerebral infarction, from a macroscopic point of view, helps the body gradually return to steady state, making it an entropy-increasing process. Our results show that, in the cerebral infarction condition, Yin and Yang are nearly equivalent and that the entropy of the metabolism network in the disease state is low. The equilibrium between Yin and Yang was broken, and the difference increased with an increase in dose. Furthermore, the entropy value of metabolism was increased and maintained at a certain level. Whether it was a metabolite expression profile or circulation of entropy, they all shifted from an ordered steady state to a disordered state. This “disordered state” may be a new harmonious state of “Yin Ping Yang Bi.”

Traditional Chinese herbs hold great promise for treating complex diseases in an integrative way (Yongxiang, 2017). However, understanding the pharmacological mechanisms of herbs at a systems and dynamics level remains a challenge. How does DHI restore the harmonious state of cerebral ischemia? We found a solution from both qualitative and quantitative viewpoints. Through qualitative analysis of metabolite transforming force patterns, a total of seven metabolites were identified, which constantly reversed in a DHI dose-dependent manner. We compared this result with module nodes. L-leucine, an overlapping metabolite of synchronous nodes identified in the DH1–DH4 full-course synchronous network module associated with constantly reversed nodes, was acquired. In addition, by using different doses for the synchronous network comparisons, we found the mechanism behind the DHI dose–response relationship (Figure 6B). Glycine-related synthesis and metabolic pathway were found to be important mechanisms for this transition state. Tetrahydrofolate is one of the important sources of glycine (Kikuchi et al., 2008). Glycine decarboxylase and serine hydroxy methyltransferase catalyze reversible folate-dependent reactions, capable of converting serine to glycine (Bruin et al., 1973). The target range expansion and the emergence of a new synchronous module may be a special pharmacological mechanism of enhanced efficacy. Glycine can inhibit damage to brain tissue caused by a glutamate neurotransmitter (Dopico et al., 2006). In the glutamate synthesis process, the glyoxylate cycle is one of the central approaches (Beeckmans, 2009). Transitional situation DH2/DH3 in our study produced something between general efficacy and better efficacy. In this state, a decrease in tetrahydrofolate results in a decrease in glycine expression, which leads to an increase in N-acetyl-L-glutamate levels due to weakening of the inhibitory ability. Although the concentration of glyoxylic acid was increased in the DH3/DH4 synchronous module, the concentration of glutamic acid was greatly reduced. This could be the molecular basis for unique therapeutic effects.

Module pharmacology is a useful tool for multitarget analysis, and the method used to identify dynamic functional modules is a critical issue (Wang et al., 2012; Wang and Wang, 2013). Synchronicity refers to a dynamic process wherein two (or more) systems adjust a given property of their motion according to a common behavior due to the interaction between them (Pecora and Carroll, 2015). Here, four DHI dose groups were used as an example to formulate a strategy to identify the dynamic synchronous module. Unlike our previous research (Wenjuan et al., 2018), this research was not limited to two targets. We utilized synchronous phenomena to find a multitarget interaction, which may help us better understand the mechanisms of dose-dependent relationships and multitarget therapies and even provide methodological support for finding the optimal dose. Meanwhile, this method can also provide a reference approach for revealing the scientific connotations of the complex pharmacological mechanisms of traditional Chinese medicine.

Despite the rigorous module pharmacology analysis of this study, there are still some shortcomings. This study considered only the expression of the node, with no network structure of modules. This makes further optimization of synchronous modules quantitative analysis procedure possible.

## Data Availability Statement

The original contributions presented in the study are included in the article/Supplementary Material, further inquiries can be directed to the corresponding author/s.

## Ethics Statement

The animal study was reviewed and approved by the Ethics Review Committee for Animal Experimentation, China Academy of Chinese Medical Sciences.

## Author Contributions

ZhoW, LZ, and YZ participated in research design. WX, JL, and BL conducted experiments. NZ, QJ, and ZhiW performed data analysis. YQ, BL, and YZ wrote and contributed to the writing of the manuscript. All authors contributed to the article and approved the submitted version.

## Conflict of Interest

ZhiW was employed by the company IBM, International Business Machines Corporation, Shanghai. The remaining authors declare that the research was conducted in the absence of any commercial or financial relationships that could be construed as a potential conflict of interest.

## Publisher’s Note

All claims expressed in this article are solely those of the authors and do not necessarily represent those of their affiliated organizations, or those of the publisher, the editors and the reviewers. Any product that may be evaluated in this article, or claim that may be made by its manufacturer, is not guaranteed or endorsed by the publisher.

## Footnotes

1
https://www.metaboanalyst.ca

## Supplementary Material

Supplementary Figure 1 Module analysis in DH1-DH4 full-course synchronous network. **(A)** Heat map of Kendall’s coefficient between 41 metabolites in DH1-DH4 full-course. The colored boxes on the right indicate the relative coefficient of the corresponding metabolite. **(B)** DH1-DH4 Synchronous network constructed by Kendall’s coefficient of concordance. Solid lines indicate positive synchronization, and dashed lines indicate negative synchronization between nodes. The thickness of the line is proportional to the Kendall’s correlation coefficient. **(C)** Three synchronous modules divided in DH1-DH4 network.

Supplementary Figure 2 Pathway enrichment analysis of targeted metabolites involved in DH1-DH4 full-course synchronous modules. **(A)** Visualization of pathways analysis. **(B)** Detailed display of each pathway.

Supplementary Figure 3 Module analysis in DH1/DH2 synchronous network. **(A)** Heat map of Kendall’s coefficient between 41 metabolites in DH1/DH2. The colored boxes on the right indicate the relative coefficient of the corresponding metabolite. **(B)** DH1/DH2 Synchronous network constructed by Kendall’s coefficient of concordance. Solid lines indicate positive synchronization, and dashed lines indicate negative synchronization between nodes. The thickness of the line is proportional to the Kendall’s correlation coefficient. **(C)** Two synchronous modules divided in DH1/DH2 network.

Supplementary Figure 4 Pathway enrichment analysis of targeted metabolites involved in DH1/DH2 synchronous modules. **(A)** Visualization of pathways analysis. **(B)** Detailed display of each pathway.

Supplementary Figure 5 Pathway enrichment analysis of targeted metabolites involved in DH2/DH3 synchronous modules. **(A)** Visualization of pathways analysis. **(B)** Detailed display of each pathway.

Supplementary Figure 6 Module analysis in DH3/DH4 synchronous network. **(A)** Heat map of Kendall’s coefficient between 41 metabolites in DH3/DH4. The colored boxes on the right indicate the relative coefficient of the corresponding metabolite. **(B)** DH3/DH4 Synchronous network constructed by Kendall’s coefficient of concordance. Solid lines indicate positive synchronization, and dashed lines indicate negative synchronization between nodes. The thickness of the line is proportional to the Kendall’s correlation coefficient. **(C)** Three synchronous modules divided in DH3/DH4 network.

Supplementary Figure 7 Pathway enrichment analysis of targeted metabolites involved in DH3/DH4 synchronous modules. **(A)** Visualization of pathways analysis. **(B)** Detailed display of each pathway.

## Abbreviations

DHI: Danhong injection
WGCNA: Weighted Correlation Network analysis
PT: positive transformation
NT: negative transformation
PA: positive add
NA: negative add
NR: negative reduce
PR: positive reduce
MCODE: Molecular Complex Detection
GO: gene ontology
KEGG: Kyoto Encyclopedia of Genes and Genomes.
