# Supplementary figures and images for: Dose-Dependent Variation of Synchronous Metabolites and Modules in a Yin/Yang Transformation Model of Appointed Ischemia Metabolic Networks
Source: Front Neurosci. 2021 Aug 20;15:645185. doi: 10.3389/fnins.2021.645185 (PMC8439200; doi:10.3389/fnins.2021.645185)

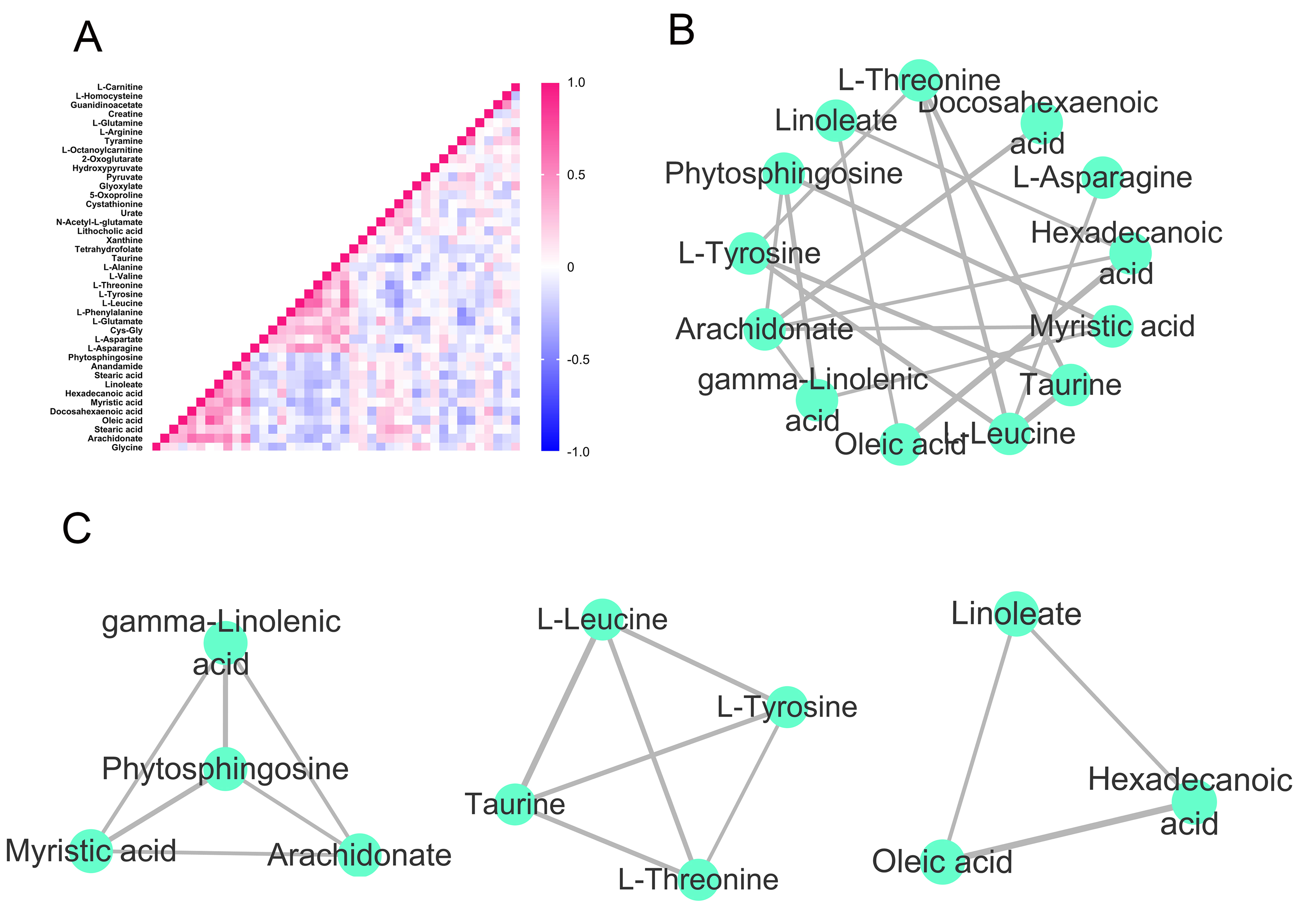

Supplement: Supplementary Figure 1 — Module analysis in DH1-DH4 full-course synchronous network. (A) Heat map of Kendall’s coefficient between 41 metabolites in DH1-DH4 full-course. The colored boxes on the right indicate the relative coefficient of the corresponding metabolite. (B) DH1-DH4 Synchronous network constructed by Kendall’s coefficient of concordance. Solid lines indicate positive synchronization, and dashed lines indicate negative synchronization between nodes. The thickness of the line is proportional to the Kendall’s correlation coefficient. (C) Three synchronous modules divided in DH1-DH4 network. [file Image_1.tif]

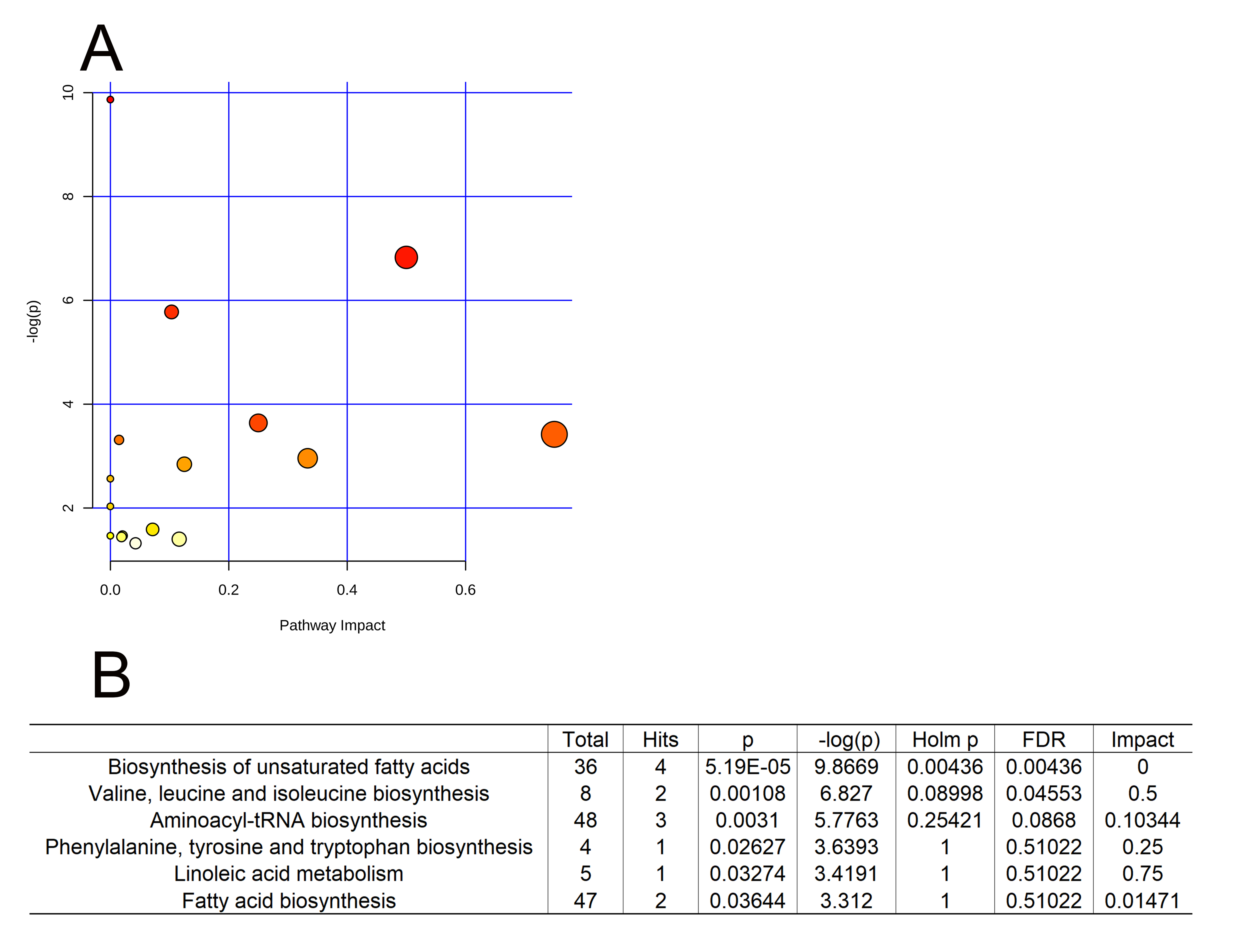

Supplement: Supplementary Figure 2 — Pathway enrichment analysis of targeted metabolites involved in DH1-DH4 full-course synchronous modules. (A) Visualization of pathways analysis. (B) Detailed display of each pathway. [file Image_2.tif]

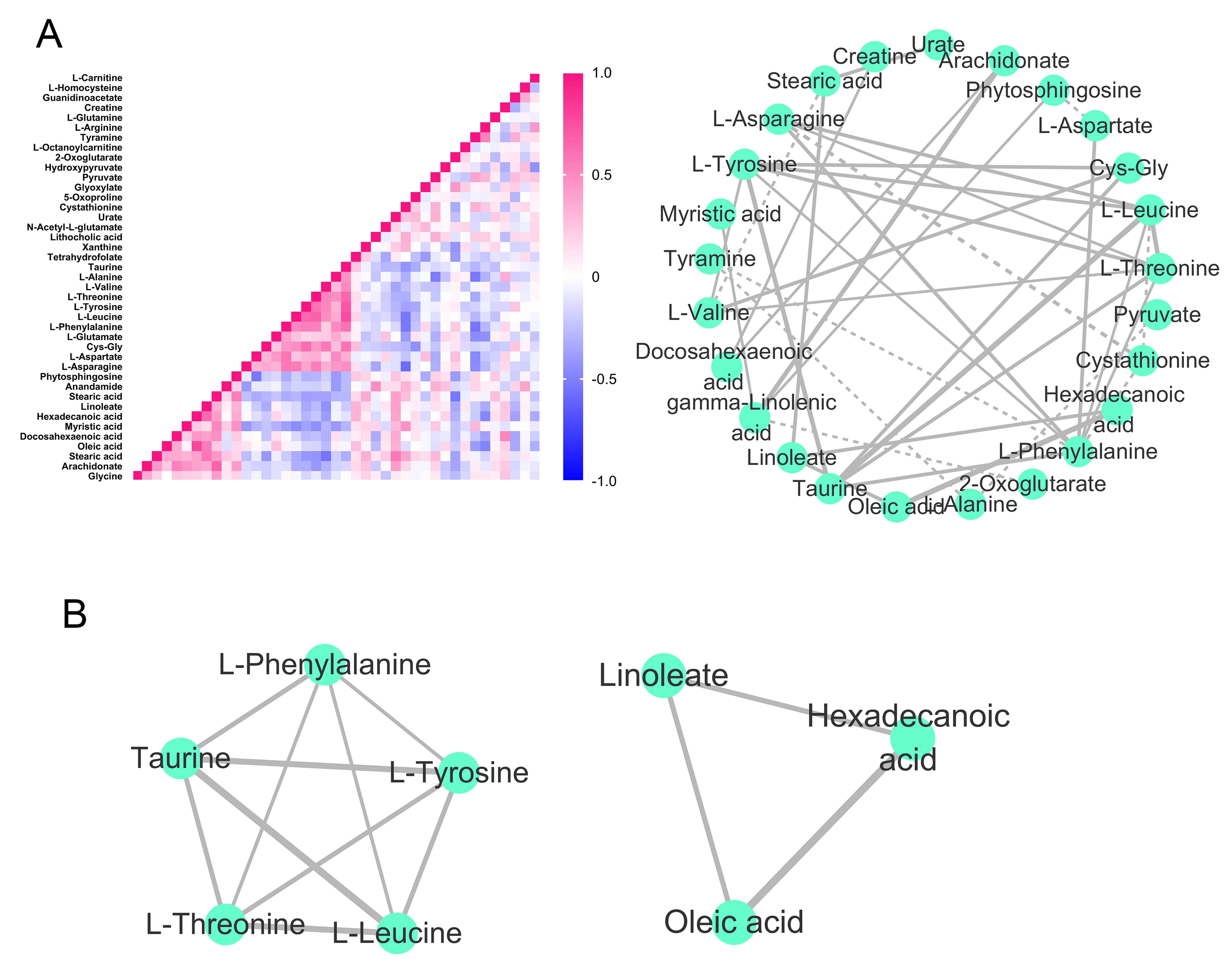

Supplement: Supplementary Figure 3 — Module analysis in DH1/DH2 synchronous network. (A) Heat map of Kendall’s coefficient between 41 metabolites in DH1/DH2. The colored boxes on the right indicate the relative coefficient of the corresponding metabolite. (B) DH1/DH2 Synchronous network constructed by Kendall’s coefficient of concordance. Solid lines indicate positive synchronization, and dashed lines indicate negative synchronization between nodes. The thickness of the line is proportional to the Kendall’s correlation coefficient. (C) Two synchronous modules divided in DH1/DH2 network. [file Image_3.tif]

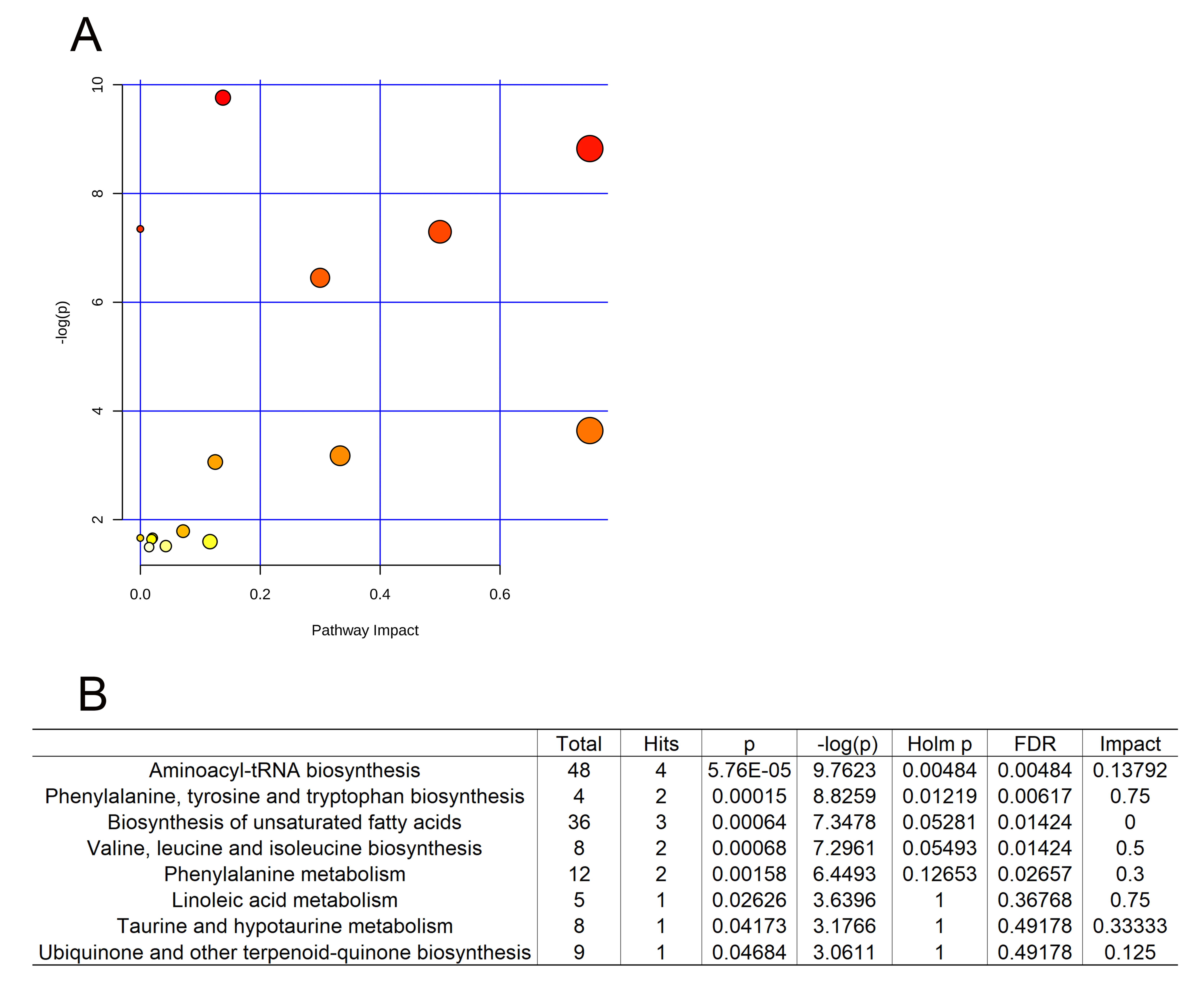

Supplement: Supplementary Figure 4 — Pathway enrichment analysis of targeted metabolites involved in DH1/DH2 synchronous modules. (A) Visualization of pathways analysis. (B) Detailed display of each pathway. [file Image_4.tif]

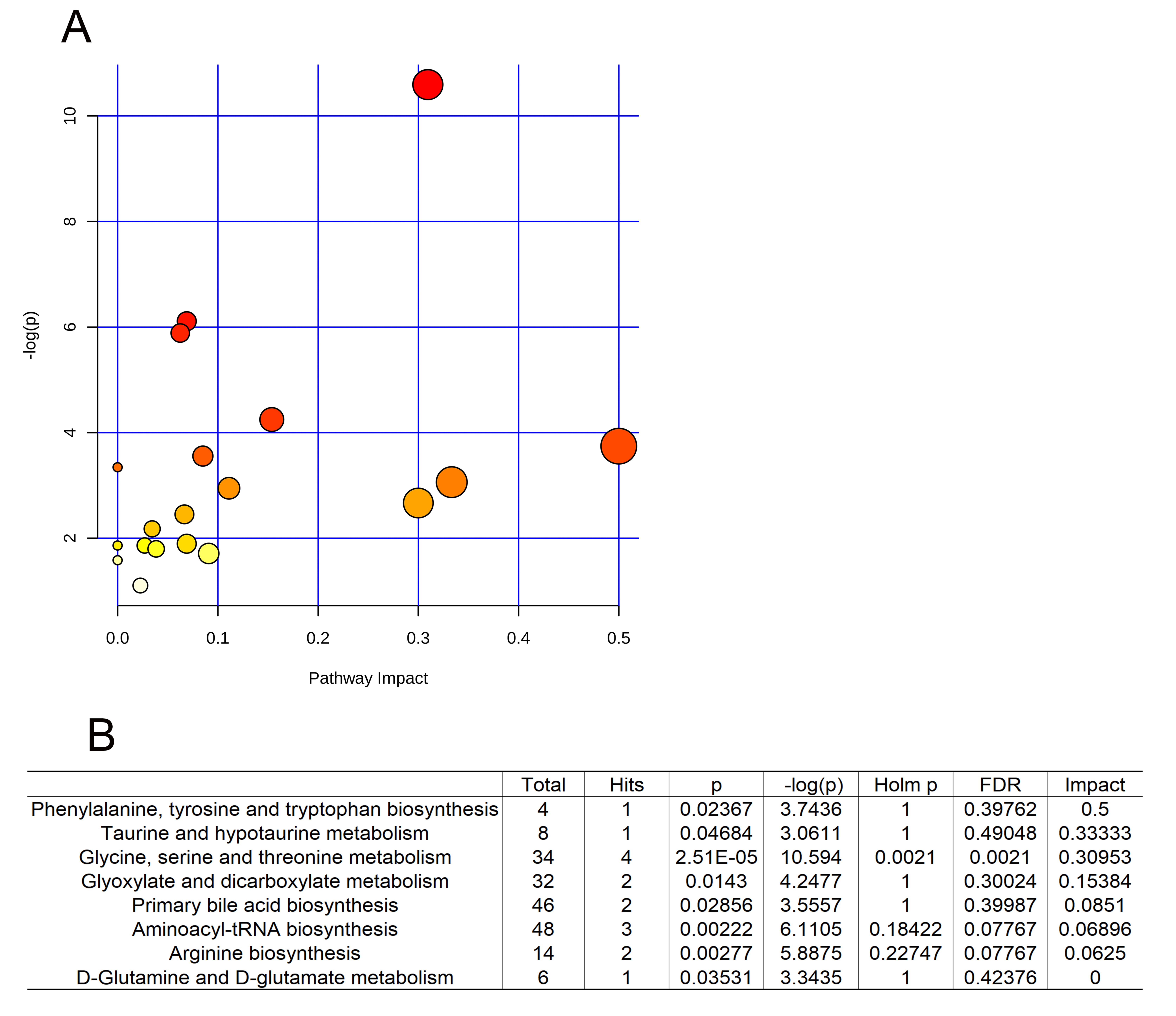

Supplement: Supplementary Figure 5 — Pathway enrichment analysis of targeted metabolites involved in DH2/DH3 synchronous modules. (A) Visualization of pathways analysis. (B) Detailed display of each pathway. [file Image_5.tif]

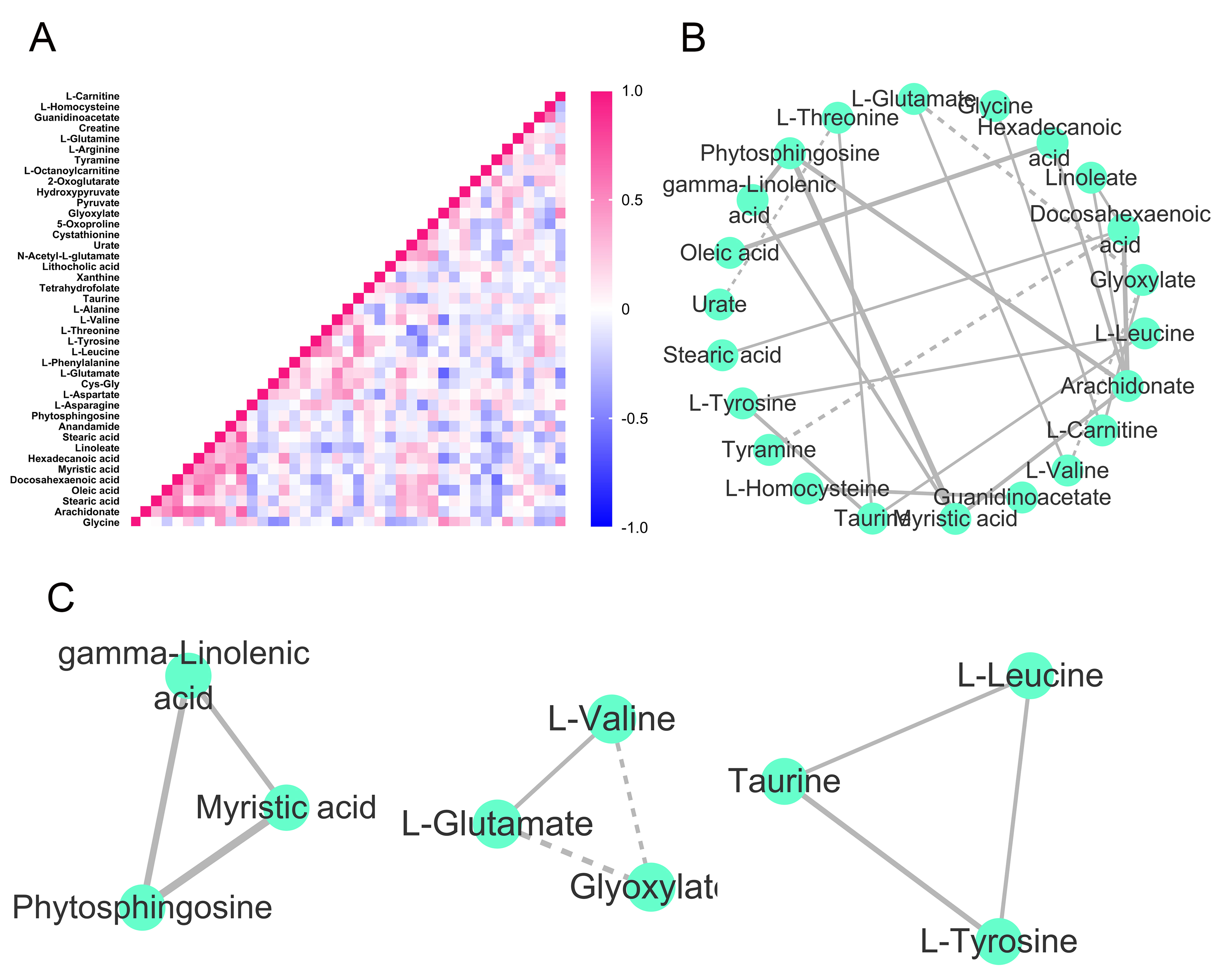

Supplement: Supplementary Figure 6 — Module analysis in DH3/DH4 synchronous network. (A) Heat map of Kendall’s coefficient between 41 metabolites in DH3/DH4. The colored boxes on the right indicate the relative coefficient of the corresponding metabolite. (B) DH3/DH4 Synchronous network constructed by Kendall’s coefficient of concordance. Solid lines indicate positive synchronization, and dashed lines indicate negative synchronization between nodes. The thickness of the line is proportional to the Kendall’s correlation coefficient. (C) Three synchronous modules divided in DH3/DH4 network. [file Image_6.tif]

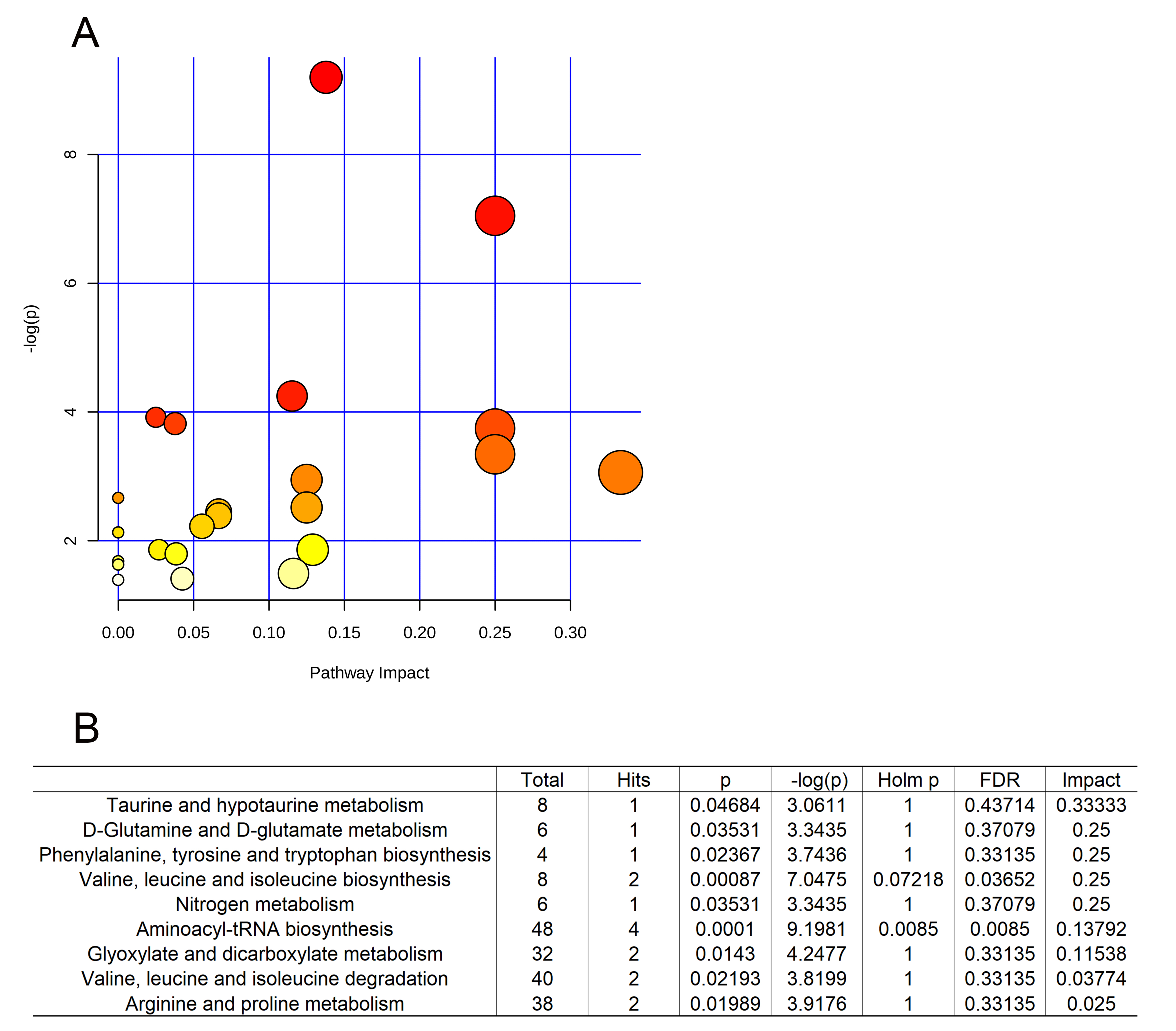

Supplement: Supplementary Figure 7 — Pathway enrichment analysis of targeted metabolites involved in DH3/DH4 synchronous modules. (A) Visualization of pathways analysis. (B) Detailed display of each pathway. [file Image_7.tif]
