# Supplementary material for: Dose-Dependent Variation of Synchronous Metabolites and Modules in a Yin/Yang Transformation Model of Appointed Ischemia Metabolic Networks
Source: Front Neurosci. 2021 Aug 20;15:645185. doi: 10.3389/fnins.2021.645185 (PMC8439200; doi:10.3389/fnins.2021.645185)
Supplement: Supplementary file 8 [file Table_1.docx]

**Table S1 Ischemia-related biomarkers analyzed based on targeted metabolomics**

| **Metabolites** | **Formula** | **KEGG** | **Mass** |
| --- | --- | --- | --- |
| ^a^L-Leucine-d3 | C_6_H_10_D_3_NO_2_ | C00123 | 135.1207 |
| L-Carnitine | C_7_H_15_NO_3_ | C00318 | 162.1125 |
| L-Homocysteine | C_4_H_9_NO_2_S | C00155 | 136.0427 |
| Guanidinoacetate | C_3_H_7_N_3_O_2_ | C00581 | 118.0611 |
| Creatine | C_4_H_9_N_3_O_2_ | C00300 | 132.0768 |
| L-Glutamine | C_5_H_10_N_2_O_3_ | C00064 | 147.0764 |
| L-Arginine | C_6_H_14_N_4_O_2_ | C00062 | 175.1190 |
| Tyramine | C_8_H_11_NO | C00483 | 138.0913 |
| L-Octanoylcarnitine | C_15_H_29_NO_4_ | C02838 | 288.2169 |
| 2-Oxoglutarate | C_5_H_6_O_5_ | C00026 | 147.0288 |
| Glycine | C_2_H_5_NO_2_ | C00037 | 76.0393 |
| ^a^Succinic acid-^13^C4 | [^13^C]_4_H_6_O_4_ | C00042 | 121.0328 |
| Hydroxypyruvate | C_3_H_4_O_4_ | C00168 | 103.0037 |
| Pyruvate | C_3_H_4_O_3_ | C00022 | 87.0088 |
| Glyoxylate | C_2_H_2_O_3_ | C00048 | 72.9931 |
| 5-Oxoproline | C_5_H_7_NO_3_ | C01879 | 128.0353 |
| Cystathionine | C_7_H_14_N_2_O_4_S | C00542 | 221.0602 |
| Urate | C_5_H_4_N_4_O_3_ | C00366 | 167.0211 |
| N-Acetyl-L-glutamate | C_7_H_11_NO_5_ | C00624 | 188.0564 |
| Lithocholic acid | C_24_H_40_O_3_ | C03990 | 421.2959 |
| Xanthine | C_5_H_4_N_4_O_2_ | C00385 | 151.0261 |
| Tetrahydrofolate | C_19_H_23_N_7_O_6_ | C00101 | 444.1637 |
| ^a^L-Leucine-d3 | C_6_H_10_D_3_NO_2_ | C00123 | 133.1062 |
| Taurine | C_2_H_7_NO_3_S | C00245 | 124.0074 |
| L-Alanine | C_3_H_7_NO_2_ | C00041 | 88.0404 |
| L-Valine | C_5_H_11_NO_2_ | C00183 | 116.0717 |
| L-Threonine | C_4_H_9_NO_3_ | C00188 | 118.0510 |
| L-Tyrosine | C_9_H_11_NO_3_ | C00082 | 180.0666 |
| L-Leucine | C_6_H_13_NO_2_ | C00123 | 130.0874 |
| L-Phenylalanine | C_9_H_11_NO_2_ | C00079 | 164.0717 |
| L-Glutamate | C_5_H_9_NO_4_ | C00025 | 146.0456 |
| Cys-Gly | C_5_H_10_N_2_O_3_S | C01419 | 177.0339 |
| L-Aspartate | C_4_H_7_NO_4_ | C00049 | 132.0302 |
| ^a^Stearic acid-d3 | C_18_H_33_D_3_O_2_ | C01530 | 286.2831 |
| Phytosphingosine | C_18_H_39_NO_3_ | C12144 | 362.2912 |
| Anandamide | C_22_H_37_NO_2_ | C11695 | 346.2752 |
| Octadecatrienoic acid | C_18_H_30_O_2_ | C06426 | 277.2173 |
| Linoleate | C_18_H_32_O_2_ | C01595 | 279.2330 |
| Hexadecanoic acid | C_16_H_32_O_2_ | C00249 | 255.2330 |
| Myristic acid | C_14_H_28_O_2_ | C06424 | 227.2017 |
| Docosahexaenoic acid | C_22_H_32_O_2_ | C06429 | 327.2330 |
| Oleic acid | C_18_H_34_O_2_ | C00712 | 281.2486 |
| Stearic acid | C_18_H_36_O_2_ | C01530 | 283.2643 |
| Arachidonate | C_20_H_32_O_2_ | C00219 | 303.2330 |
